# Supplementary material for: Herbal medicine (Hyeolbuchukeo-tang or Xuefu Zhuyu decoction) for treating primary dysmenorrhoea: protocol for a systematic review of randomised controlled trials
Source: BMJ Open. 2017 Jun 15;7(6):e015056. doi: 10.1136/bmjopen-2016-015056 (PMC5623393; doi:10.1136/bmjopen-2016-015056)
Supplement: Supplementary material [file bmjopen-2016-015056supp001.pdf]

Supplementary 1 Search strategies

[MEDLINE(Pubmed) Search Strategy]

#1 "Menstruation Disturbances"[Mesh]

#2 Dysmenorrhea [TIAB]

#3 Pain, Menstrual [TIAB]

#4 Menstrual Pain [TIAB]

#5 Menstrual Pains [TIAB]

#6 Pains, Menstrual [TIAB]

#7 Menstruation, Painful [TIAB]

#8 Menstruations, Painful [TIAB]

#9 Painful Menstruation [TIAB]

#10 Painful Menstruations [TIAB]

#11 Period pain [TIAB]

#12 Painful period [TIAB]

#13 Cramps [TIAB]

#14 Menstrual Disorder [TIAB]

#15 Pelvic pain [TIAB]

#16 1-15/or

#17 Xuefu Zhuyu [TIAB]

#18 “Xuefu Zhuyu Granule” [TIAB]

#19 “Xuefu Zhuyu Decoction” [TIAB]

#20 “Xuefu Zhuyu Formula” [TIAB]

#21 “Xuefu Zhuyu Tang” [TIAB]

#22 Xuefu Zhuyu Capsule [TIAB]

#23 “Xuefu Zhuyu Pill” [TIAB]

#24 “Xuefu Zhuyu Tablet” [TIAB]

#25 “Xuefu Zhuyu Oral Liquid” [TIAB]

#26 Xue fu Zhu yu [TIAB]

#27 “Xue fu Zhu yu Granule” [TIAB]

#28 “Xue fu Zhu yu Decoction” [TIAB]

#29 “Xue fu Zhu yu Formula” [TIAB]

#30 “Xue fu Zhu yu Tang” [TIAB]

#31 Xue fu Zhu yu Capsule [TIAB]

#32 “Xue fu Zhu yu Pill” [TIAB]

#33 “Xue fu Zhu yu Tablet” [TIAB]

#34 “Xue fu Zhu yu Oral Liquid” [TIAB]

#35 “Hyeolbuchukeo-tang” [TIAB]

#36 “Hyulbuchuko-tang” [TIAB]

#37 “Hyulboochucke-tang” [TIAB]

#38 “Hyulbuchookau-tang” [TIAB]

#39 17-38/or

#40 #16 and #39

[CNKI Search Strategy]

#1 痛经 OR 原发性痛经 OR 月经失调 OR 月经困难 OR 月经紊乱 OR 经行腹痛 OR 经痛 OR 月经痛 OR 痛性痉挛 OR 骨盆痛

Search in result

#2 血府逐瘀 OR 血府逐瘀汤 OR 血府逐瘀颗粒 OR血府逐瘀方 OR 血府逐瘀汤 OR

血府逐瘀胶囊 OR 血府逐瘀丸 OR 血府逐瘀片
