# Supplementary material for: Herbal medicine (Hyeolbuchukeo-tang or Xuefu Zhuyu decoction) for treating primary dysmenorrhoea: protocol for a systematic review of randomised controlled trials
Source: BMJ Open. 2017 Jun 15;7(6):e015056. doi: 10.1136/bmjopen-2016-015056 (PMC5623393; doi:10.1136/bmjopen-2016-015056)
Supplement: Author's manuscript [file bmjopen-2016-015056.draft_revisions.pdf]

BMJ Open

Herbal medicine (Hyeolbuchukeo-tang or Xuefu Zhuyu decoction) for treating primary dysmenorrhoea: a protocol for systematic review of randomised controlled trials

Journal:	*BMJ Open*
Manuscript ID	bmjopen-2016-015056
Article Type:	Protocol
Date Submitted by the Author:	05-Nov-2016
Complete List of Authors:	Jo, Junyoung; Conmaul Hospital of Korean Medicine, Korean Obstetrics and Gynecology<br>Leem, Jungtae; Kyung Hee University, Department of Clinical Korean Medicine<br>Lee, Jin Moo; Kyung Hee University Korean Medicine Hospital at Gangdong , Department of Korean Obstetrics & Gynecology<br>PARK, KYOUNG SUN; Kyung Hee University, Korean Medicine Obstetrics & Gynecology
**Primary Subject Heading**:	Obstetrics and gynaecology
Secondary Subject Heading:	Complementary medicine, Evidence based practice
Keywords:	dysmenorrhoea, systematic review, Herbal medicine < THERAPEUTICS, protocol

SCHOLARONE™ Manuscripts

For peer review only

Herbal medicine (Hyeolbuchukeo-tang or Xuefu Zhuyu decoction) for treating primary dysmenorrhoea: a protocol for systematic review of randomised controlled trials

Junyoung Jo[1 †], Jungtae Leem[2,3 †], Jin Moo Lee[4], Kyoung Sun Park[4*]

[1]Department of Korean Obstetrics & Gynecology, Conmaul Hospital, Seoul, Republic of Korea

[2]Korean Medicine Clinical Trial Center, Kyung Hee University Korean Medicine Hospital, Seoul, South Korea

[3]Department of Clinical Korean Medicine, Graduate School, Kyung Hee University, 26 Kyungheedae-ro, Dongdaemun-gu, Seoul, 02447, South Korea

[4]Department of Korean Obstetrics & Gynecology, Kyung Hee University Korean Medicine Hospital at Gangdong, Seoul, Republic of Korea

† equal contributors

* Correspondence: Kyung-Sun Park, M.D. (DKM), PhD

Department of Korean Obstetrics & Gynecology

Kyung Hee University Korean Medicine Hospital at Gangdong

Seoul, Republic of Korea

Tel: 82-2-440-6146

Fax: 82-2- 440-7143

E-mail: lovepks0116@gmail.com

For peer review only

ABSTRACT

Introduction:

Primary dysmenorrhoea is menstrual pain without pelvic pathology and is the most common gynaecologic condition in women. Xuefu Zhuyu decoction (XZD) or Hyeolbuchukeo-tang, a traditional herbal formula, has been used as a treatment for primary dysmenorrhoea. The purpose of this study is to assess the current published evidence regarding XZD as treatment for primary dysmenorrhoea.

Methods and analysis:

The following databases will be searched from their inception until October 2016: MEDLINE (via PubMed), Allied and Complementary Medicine Database (AMED), EMBASE, The Cochrane Library, six Korean Medical Databases (Korean Studies Information Service System, DBPIA, Oriental Medicine Advanced Searching Integrated System, Research Information Service System, Korea Med, and the Korean Traditional Knowledge Portal), three Chinese Medical Databases [China National Knowledge Infrastructure (CNKI), Wan Fang Database, and Chinese Scientific Journals Database (VIP)], and one Japanese medical database (CiNii). Randomised clinical trials (RCTs) that will be included in this systematic review comprise those that used XZD or modified XZD. The control groups in the RCTs include no treatment, placebo, conventional medication, or other treatments. Trials testing XZD as an adjunct to other treatments, as well as studies where the control group received the same treatment as the intervention group will be also included. Data extraction and risk of bias assessments will be performed by two independent reviewers. The risk of bias will be assessed with the Cochrane risk of bias tool. All statistical analyses will be conducted using Review Manager software (RevMan V.5.3.0).

Ethics and dissemination:

This systematic review will be published in a peer-reviewed journal. The review will also be disseminated electronically and in print. The review will benefit patients and practitioners in the fields of traditional and conventional medicine.

Trial registration number: CRD42016050447 in PROSPERO 2016

For peer review only

Strength and limitations of the present study protocol

- Our review will provide useful and novel information for patients, policymakers, and practitioners.
- To avoid language bias, the Chinese, Korean, and Japanese databases will be searched.
- Our systematic review will describe a comprehensive and objective assessment of the safety and effectiveness of Hyeolbuchukeo-tang/Xuefu Zhuyu decoction as treatment for primary dysmenorrhoea patients.
- We will assess the methodological and reporting quality of included studies with CONSORT extension for herbal medicine.

1. Introduction

Primary dysmenorrhoea is a common complaint that refers to painful menstrual cramps in the lower abdominal region during menstruation in the absence of an identifiable pathological condition among menstruating women.[1] Due to the different definitions of the condition, and the lack of standard methods for assessing the severity of dysmenorrhoea, prevalence estimates vary between 45 and 95% of menstruating women.[2] Dysmenorrheic pain has been reported to be the primary cause of recurrent short-term school or work absenteeism among young women of childbearing age.[3] Further, dysmenorrheic pain has an immediate negative impact on quality of life, for up to a few days every month. Women with primary dysmenorrhoea have a significantly reduced quality of life, poorer mood, and poorer sleep quality during menstruation compared with women who do not report dysmenorrhoea.[3]

Non-steroidal anti-inflammatory drugs (NSAIDs) are considered the primary treatment for primary dysmenorrhoea but the quality of the evidence is low mainly due to poor reporting of study methods. In addition, NSAIDs commonly cause adverse effects, including indigestion, headaches, and drowsiness.[4] Therefore, many women also seek alternative therapies to manage their menstrual discomfort including heating pads for cramps, transcutaneous electric nerve stimulation, Chinese herbal medicine (CHM), and acupuncture.[3 5-7] A Cochrane review suggested that CHM was promising for managing primary dysmenorrhoea, although the quality of the included studies was poor.[5] However, the review included all types of CHM and is outdated, requiring another study that focuses on a specific type of CHM.

In traditional Chinese Medicine or Korean Medicine, the main factor causing menstrual abdominal pain is

For peer review only

blood stagnation.[8] Xuefu Zhuyu decoction (XZD) or Hyeolbuchukeo-tang was the most frequent formula used in the blood stasis researches in Korea.[9] Several systematic reviews regarding other CHM such as Danggui Shaoyao San[10], Shaofu Zhuyu decoction[11] or Gyejibongneyonghwan[12] have already been published or planned. However, no systematic review regarding XZD in primary dysmenorrhoea has been planned or published yet. Therefore, in this review, we will investigate current evidence related to the effectiveness of XZD or Hyeolbuchukeo-tang, a traditional herbal formula, as a treatment for primary dysmenorrhoea.

2. Materials and Methods

Study registration

The protocol for this systematic review has been registered on PROSPERO 2016 under the number CRD42016050447.

Data sources

The following databases will be searched from inception to October 2016: Medline (via PubMed), EMBASE (via OVID), the Cochrane Central Register of Controlled Trials (CENTRAL), Allied and Complementary Medicine Database (AMED) and Cumulative Index to Nursing and Allied Health Literature (CINAHL). We will also search six Korean medical databases [Oriental Medicine Advanced Searching Integrated System (OASIS), Korean Traditional Knowledge Portal (KTKP), Korean Studies Information Service System (KISS), Research Information Service System (RISS), KoreaMed, and DBpia], three Chinese databases [China National Knowledge Infrastructure Database (CNKI), Wanfang, and Chinese Scientific Journals Database (VIP)], and one Japanese medical database (CiNii). We will also search conference proceedings of relevant journals and conduct hand searching. Clinical trial registries will also be searched. The search term will be composed of the disease term part (e.g., dysmenorrhoea, menstrual pain, painful menstruation, period pain, painful period, cramps, menstrual disorder, pelvic pain) and the intervention term part (e.g., Xuefu Zhuyu granule/decoction/formula/tang/capsule/pill/tablet).

The search strategies that will be applied to the Medline database and CNKI are presented in online supplements 1 and 2. Similar search strategies will be applied to the other databases. Study selection will be documented and summarized in a PRISMA-compliant flow chart (http://www.prisma-statement.org) (figure 1).[13]

Types of study

All prospective randomised controlled trials (RCTs) will be included

For peer review only

Type of participants

Patients with primary dysmenorrhoea will be considered in the systematic review. Dysmenorrhoea secondary to other pathologies such as uterine myoma, endometriosis, or infection will not be included in this review.

Type of interventions

Randomised studies of XZD formula as the sole treatment or as an adjunct to other treatments will be included. Studies where the control group received the same treatment as the intervention group will also be included. Trials comparing XZD formula with any type of control intervention will also be included. Control group intervention could be placebo XZD, no treatment, conventional medication, or other treatments. No language restrictions will be imposed. Hard copies of all articles will be obtained and read in full text.

Data extraction

Two authors (JJ and J Leem) will perform the data extraction and quality assessment using a predefined data extraction form. The form includes information pertaining to first author, study design, language of publication, country where the trial was conducted, clinical setting, diagnostic criteria, disease duration, number of participants allocated to each group, drop out number, treatment duration, dosage of XZD, pattern identification of the participants' comparison groups, outcome, outcome results, follow-up periods, adverse events associated with XZD, and composition of XZD. When studies report outcomes at more than one time point, a similar measurement point in other studies will be obtained for analysis. Any disagreement among the authors will be resolved by discussion among all of the authors. When the data are insufficient or ambiguous, JL will contact the corresponding authors by e-mail or telephone to request additional information or clarification.

Assessment of risk of bias in included studies

The risk of bias will be assessed using the assessment tool for the risk of bias from the Cochrane Handbook V.5.1.0, which includes random sequence generation, allocation concealment, blinding of the participants and personnel, blinding of the outcome assessments, incomplete outcome data, selective reporting, and other sources of bias.[14] Our review will use 'L', 'U', and 'H' to indicate the results of the assessments: 'L' indicates a low risk of bias, 'U' indicates that the risk of bias was unclear, and 'H' indicates a high risk of bias. Disagreements will be resolved by discussion between all of the authors. When disagreements regarding selection cannot be

For peer review only

resolved through discussion, the arbiter (KP) will make the final decision.

Outcome measures

Primary outcomes

- Change in symptoms as indicated on a 100 mm visual analogue scale (VAS)
- Response rate: an overall reduction in symptoms (menstruation-related symptoms including dysmenorrhoea)

As most Chinese trials report outcomes based on a categorical assessment (e.g. 'markedly improved', 'improved', 'slightly better', or 'no effect'), all patients except those assessed with 'no effect' on pain relief will be counted as having reduced pain. For example, if a treatment group of 100 women are measured for intensity of pain using markedly improved (n = 30), moderately improved (n = 40), slightly better (n = 20), or no reduction (n=10), then the number of women who report any reduction (n = 90) will be considered as the responder group and included in the meta-analysis as having experienced a reduction in pain (n = 90/100).

Secondary outcomes

- Quality of life as measured using validated questionnaires
- Adverse events

Data synthesis

Statistical analyses will be performed with the Review Manager program (Version 5.3 Copenhagen: The Nordic Cochrane Centre, The Cochrane Collaboration, 2014). Trials will be combined according to the type of intervention and type of outcome measure and/or control. Data will be pooled and expressed as mean differences (MD) or standardized mean difference (SMD) for continuous outcomes and risk ratio (RR) for dichotomous outcomes with 95% confidence intervals (CI) using fixed or random-effects models.

Dealing with missing data

As much as possible, we will analyze the data using an intention-to-treat (ITT) basis, and we will attempt to obtain missing data from the original investigators. If these attempts are not successful, we will not impute data for missing data; we will analyze only the available data.

For peer review only

Assessment and investigation of heterogeneity

Heterogeneity among studies will be assessed using χ^2 (chi-squared) test with a significance level of $P < 0.1$ and I^2 statistic.[15] The I^2 statistic indicates the proportion of variability among trials that is not explained by chance alone and we consider an I^2 value > 50% to indicate a substantial heterogeneity.[15,16] If substantial heterogeneity is detected, we will explore sources of heterogeneity by performing subgroup analysis. If some factors (e.g., lack of included trials, large methodological and/or clinical difference among trials) are found, we will not conduct subgroup analysis or data synthesis, but report a narrative description of the included studies. Subgroup analyses will be attempted according to type of control (e.g., kind of medicine), taking into consideration the characteristics of the included studies.

Subgroup analysis

If a sufficient number of subgroup studies exist, subgroup analysis will be conducted to identify heterogeneity between subgroups. Subgroup analysis criteria are as follows: 1) duration of herbal medicine treatment; 2) type of control intervention: placebo XZD, no treatment, or western medication; and 3) duration or severity of primary dysmenorrhoea.

Sensitivity analysis

Methodological and reporting quality of included studies will be assessed by the consolidated standards of reporting trials (CONSORT) extension for herbal interventions.[17] To identify the robustness of the meta-analysis result, sensitivity analysis will be conducted after excluding low quality trials. We will compare original and sensitivity meta-analysis results.

Assessment of reporting biases

When there are more than 10 trials in the analysis, reporting biases such as publication bias will be assessed by funnel plots. If asymmetry is suggested by a visual inspection, we will perform exploratory analyses using Egger's method.[15]

3. Discussion

The purpose of our review is to assess the effectiveness and safety of XZD in women with primary dysmenorrhoea. Several systematic reviews of CHM have already been published.[5 10 11] Even though XZD is

For peer review only

frequently used in primary dysmenorrhoea[18], no systematic reviews on the effects of XZD formula on primary dysmenorrhoea have been published. This systematic review will provide a summary of the current evidence related to the effectiveness of XZD formula for the treatment of primary dysmenorrhoea. In particular, we will identify subtypes that are particularly useful for specific subgroups according to TCM theory or TCM pattern identification. We will also identify a range of dosages and modifications used to improve effectiveness in full review of this protocol. Detailed information of clinical trial regimens of XZD in primary dysmenorrhoea will give insight to researchers who are planning XZD clinical trials in primary dysmenorrhoea. We also anticipate finding predicting factors of treatment response by subgroup analysis. This evidence will also be useful to medical practitioners and patients in the field of women health care.

Reference

1. Dawood MY. Primary dysmenorrhoea: advances in pathogenesis and management. *Obstetrics and gynecology* 2006;108(2):428-41. doi: 10.1097/01.AOG.0000230214.26638.0c [published Online First: 2006/08/02]
2. Proctor M, Farquhar C. Diagnosis and management of dysmenorrhoea. *BMJ (Clinical research ed)* 2006;332(7550):1134-8. doi: 10.1136/bmj.332.7550.1134 [published Online First: 2006/05/13]
3. Iacovides S, Avidon I, Baker FC. What we know about primary dysmenorrhoea today: a critical review. *Human reproduction update* 2015;21(6):762-78. doi: 10.1093/humupd/dmv039 [published Online First: 2015/09/09]
4. Marjoribanks J, Ayeleke RO, Farquhar C, et al. Nonsteroidal anti-inflammatory drugs for dysmenorrhoea. *The Cochrane database of systematic reviews* 2015(7):Cd001751. doi: 10.1002/14651858.CD001751.pub3 [published Online First: 2015/08/01]
5. Zhu X, Proctor M, Bensoussan A, et al. Chinese herbal medicine for primary dysmenorrhoea. *The Cochrane database of systematic reviews* 2008(2):Cd005288. doi: 10.1002/14651858.CD005288.pub3 [published Online First: 2008/04/22]
6. Smith CA, Armour M, Zhu X, et al. Acupuncture for dysmenorrhoea. *The Cochrane database of systematic reviews* 2016;4:Cd007854. doi: 10.1002/14651858.CD007854.pub3 [published Online First: 2016/04/19]
7. Igwea SE, Tabansi-Ochuogu CS, Abaraogu UO. TENS and heat therapy for pain relief and quality of life improvement in individuals with primary dysmenorrhoea: A systematic review. *Complementary therapies in clinical practice* 2016;24:86-91. doi: 10.1016/j.ctcp.2016.05.001 [published Online First: 2016/08/10]
8. Park JS, Park S, Cheon CH, et al. Effects and safety of gyejibongnyeong-hwan on

For peer review only

dysmenorrhoea caused by blood stagnation: a randomised controlled trial. *Evidence-based complementary and alternative medicine : eCAM* 2013;2013:424730. doi: 10.1155/2013/424730 [published Online First: 2013/11/06]

9. Park B, You S, Jung J, et al. Korean studies on blood stasis: an overview. *Evidence-based complementary and alternative medicine : eCAM* 2015;2015:316872. doi: 10.1155/2015/316872 [published Online First: 2015/03/31]

10. Lee HW, Jun JH, Kil KJ, et al. Herbal medicine (Danggui Shaoyao San) for treating primary dysmenorrhoea: A systematic review and meta-analysis of randomised controlled trials. *Maturitas* 2016;85:19-26. doi: 10.1016/j.maturitas.2015.11.013 [published Online First: 2016/02/10]

11. Lee H, Choi TY, Myung CS, et al. Herbal medicine (Shaofu Zhuyu decoction) for treating primary dysmenorrhoea: A systematic review of randomised clinical trials. *Maturitas* 2016;86:64-73. doi: 10.1016/j.maturitas.2016.01.012 [published Online First: 2016/02/29]

12. Lee JA, Park S, Jung J, et al. Herbal medicine (Gyejibongneyong-hwan) for treating primary dysmenorrhoea: a protocol for a systematic review of randomised controlled trials. *BMJ open* 2016;6(9):e011071. doi: 10.1136/bmjopen-2016-011071 [published Online First: 2016/09/30]

13. Liberati A, Altman DG, Tetzlaff J, et al. The PRISMA statement for reporting systematic reviews and meta-analyses of studies that evaluate health care interventions: explanation and elaboration. *Annals of internal medicine* 2009;151(4):W65-94. [published Online First: 2009/07/23]

14. Higgins JPT, Altman DG. "Chapter 8: assessing risk of bias in included studies," in Cochrane Handbook for Systematic Reviews of Interventions Version 5.1.0, J. Higgins and S. Green, Eds., The Cochrane Collaboration, 2011, http://www.cochrane-handbook.org. 2011 [

15. Deeks JJ, Higgins JPT, Altman DG. "Chapter 9: analyzing data and undertaking meta-analyses," in Cochrane Handbook for Systematic Reviews of Interventions Version 5.1.0, J. Higgins and S. Green, Eds., The Cochrane Collaboration, 2011, http://www.cochrane-handbook.org. 2011 [

16. Higgins JP, Thompson SG. Quantifying heterogeneity in a meta-analysis. *Stat Med* 2002;21(11):1539-5158.

17. Gagnier JJ, Boon H, Rochon P, et al. Recommendations for reporting randomised controlled trials of herbal interventions: Explanation and elaboration. *Journal of clinical epidemiology* 2006;59(11):1134-49. doi: 10.1016/j.jclinepi.2005.12.020 [published Online First: 2006/10/10]

18. Jia W, Wang X, Xu D, et al. Common traditional Chinese medicinal herbs for dysmenorrhoea. *Phytotherapy research : PTR* 2006;20(10):819-24. doi: 10.1002/ptr.1905 [published Online First: 2006/07/13]

For peer review only

AUTHORS′ CONTRIBUTIONS

The study was conceptualized by JJ. The protocol was drafted by JJ and J Leem. The search strategy was developed by J Leem and JJ. J Lee and KP revised the manuscript. J Leem submitted the manuscript for publication. All authors have read and approved the final manuscript.

FUNDING AND ACKNOWLEDGEMENTS

This study is supported by the Traditional Korean Medicine R&D program that is funded by the Ministry of Health & Welfare through the Korea Health Industry Development Institute (KHIDI, grant HB16C0018).

COMPETING INTERESTS

The authors declare no competing interests.

PROVENANCE AND PEER REVIEW

Not commissioned; externally peer reviewed.

For peer review only

Figure 1 PRISMA flow diagram

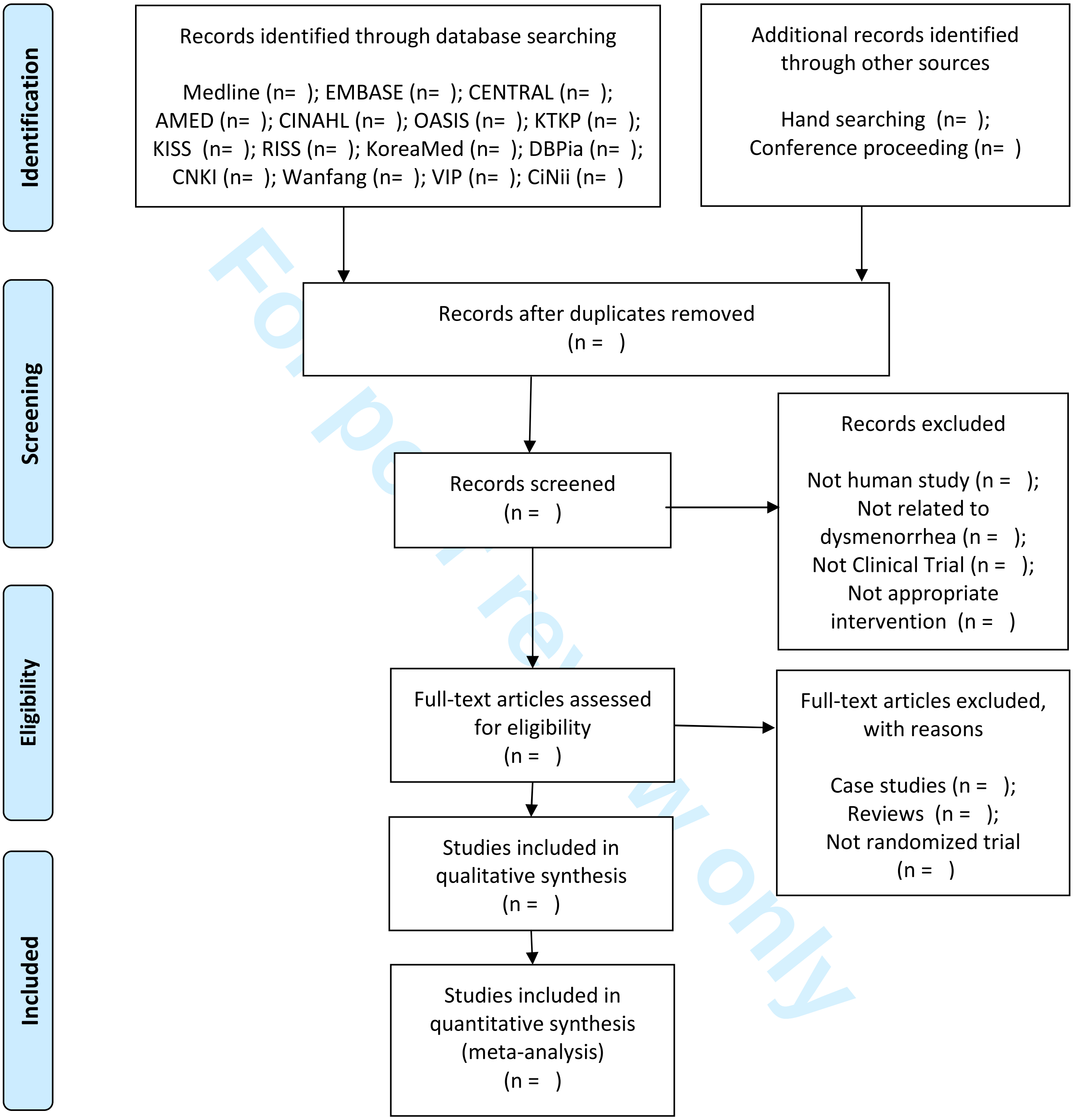

From: Moher D, Liberati A, Tetzlaff J, Altman DG, The PRISMA Group (2009). *P*referred *R*eporting *I*tems for *S*ystematic Reviews and *M*eta-*A*nalyses: The PRISMA Statement. PLoS Med 6(6): e1000097. doi:10.1371/journal.pmed1000097

For more information, visit www.prisma-statement.org.

For peer review only

PRISMA-P 2015 Checklist

This checklist has been adapted for use with protocol submissions to *Systematic Reviews* from Table 3 in Moher D et al: Preferred reporting items for systematic review and meta-analysis protocols (PRISMA-P) 2015 statement. *Systematic Reviews* 2015 **4**:1

Section/topic	#	Checklist item	Information reported		Line number(s)
			Yes	No	
**ADMINISTRATIVE INFORMATION**					
**Title**					
Identification	1a	Identify the report as a protocol of a systematic review	☐		1-2
Update	1b	If the protocol is for an update of a previous systematic review, identify as such			Not update for previous review
**Registration**	2	If registered, provide the name of the registry (e.g., PROSPERO) and registration number in the Abstract	☐		50
**Authors**					
Contact	3a	Provide name, institutional affiliation, and e-mail address of all protocol authors; provide physical mailing address of corresponding author	☐		4-20
Contributions	3b	Describe contributions of protocol authors and identify the guarantor of the review	☐		274-276
**Amendments**	4	If the protocol represents an amendment of a previously completed or published protocol, identify as such and list changes; otherwise, state plan for documenting important protocol amendments			Not amendment for previous review
**Support**					
Sources	5a	Indicate sources of financial or other support for the review	☐		279-280
Sponsor	5b	Provide name for the review funder and/or sponsor	☐		279-280
Role of sponsor/funder	5c	Describe roles of funder(s), sponsor(s), and/or institution(s), if any, in developing the protocol			No roles exist
**INTRODUCTION**					

BioMed Central
The Open Access Publisher

For peer review only

Section/topic	#	Checklist item	Information reported		Line number(s)
			Yes	No	
**Rationale**	6	Describe the rationale for the review in the context of what is already known	☐		72-86
**Objectives**	7	Provide an explicit statement of the question(s) the review will address with reference to participants, interventions, comparators, and outcomes (PICO)	☐		109-156
**METHODS**					
**Eligibility criteria**	8	Specify the study characteristics (e.g., PICO, study design, setting, time frame) and report characteristics (e.g., years considered, language, publication status) to be used as criteria for eligibility for the review	☐		109-121
**Information sources**	9	Describe all intended information sources (e.g., electronic databases, contact with study authors, trial registers, or other grey literature sources) with planned dates of coverage	☐		93-108
**Search strategy**	10	Present draft of search strategy to be used for at least one electronic database, including planned limits, such that it could be repeated	☐		93-108
STUDY RECORDS					
Data management	11a	Describe the mechanism(s) that will be used to manage records and data throughout the review	☐		123-132
Selection process	11b	State the process that will be used for selecting studies (e.g., two independent reviewers) through each phase of the review (i.e., screening, eligibility, and inclusion in meta-analysis)	☐		123-132
Data collection process	11c	Describe planned method of extracting data from reports (e.g., piloting forms, done independently, in duplicate), any processes for obtaining and confirming data from investigators	☐		123-132
**Data items**	12	List and define all variables for which data will be sought (e.g., PICO items, funding sources), any pre-planned data assumptions and simplifications	☐		123-132
**Outcomes and prioritization**	13	List and define all outcomes for which data will be sought, including prioritization of main and additional outcomes, with rationale	☐		144-156
**Risk of bias in individual studies**	14	Describe anticipated methods for assessing risk of bias of individual studies, including whether this will be done at the outcome or study level, or both; state how this information will be used in data synthesis	☐		134-141
DATA					
**Synthesis**	15a	Describe criteria under which study data will be quantitatively synthesized	☐		158-163
	15b	If data are appropriate for quantitative synthesis, describe planned summary measures, methods of handling data, and methods of combining data from studies, including any planned exploration	☐		170-178

BioMed Central The Open Access Publisher

For peer review only

Section/topic	#	Checklist item	Information reported		Line number(s)
			Yes	No	
		of consistency (e.g., I^2, Kendall's tau)			
	15c	Describe any proposed additional analyses (e.g., sensitivity or subgroup analyses, meta-regression)	☐		180-190
	15d	If quantitative synthesis is not appropriate, describe the type of summary planned	☐		170-178
**Meta-bias(es)**	16	Specify any planned assessment of meta-bias(es) (e.g., publication bias across studies, selective reporting within studies)	☐		192-195
**Confidence in cumulative evidence**	17	Describe how the strength of the body of evidence will be assessed (e.g., GRADE)		☐	Not Applicable

For peer review only

BioMed Central
The Open Access Publisher

Appendix 1 : MEDLINE(Pubmed) Search Strategy

#1 "Menstruation Disturbances"[Mesh]

#2 Dysmenorrhea [TIAB]

#3 Pain, Menstrual [TIAB]

#4 Menstrual Pain [TIAB]

#5 Menstrual Pains [TIAB]

#6 Pains, Menstrual [TIAB]

#7 Menstruation, Painful [TIAB]

#8 Menstruations, Painful [TIAB]

#9 Painful Menstruation [TIAB]

#10 Painful Menstruations [TIAB]

#11 Period pain [TIAB]

#12 Painful period [TIAB]

#13 Cramps [TIAB]

#14 Menstrual Disorder [TIAB]

#15 Pelvic pain [TIAB]

#16 1-15/or

#17 Xuefu Zhuyu [TIAB]

#18 “Xuefu Zhuyu Granule” [TIAB]

#19 “Xuefu Zhuyu Decoction” [TIAB]

#20 “Xuefu Zhuyu Formula” [TIAB]

#21 “Xuefu Zhuyu Tang” [TIAB]

#22 Xuefu Zhuyu Capsule [TIAB]

#23 “Xuefu Zhuyu Pill” [TIAB]

#24 “Xuefu Zhuyu Tablet” [TIAB]

For peer review only

#25 “Xuefu Zhuyu Oral Liquid” [TIAB]

#26 “Hyeolbuchukeo-tang” [TIAB]

#27 “Hyulbuchuko-tang” [TIAB]

#28 “Hyulboochucke-tang” [TIAB]

#29 “Hyulbuchookau-tang” [TIAB]

#30 17-29/or

#31 #16 and #30

For peer review only

Appendix 2 : CNKI Search Strategy

#1 痛经 OR 原发性痛经 OR 月经失调 OR 月经困难 OR 月经紊乱 OR 经行腹痛 OR 经痛 OR 月经痛 OR 痛性痉挛 OR 骨盆痛

Search in result

#2 血府逐瘀 OR 血府逐瘀汤 OR 血府逐瘀颗粒 OR血府逐瘀方 OR 血府逐瘀汤 OR 血府逐瘀胶囊 OR 血府逐瘀丸 OR 血府逐瘀片

For peer review only

BMJ Open

Herbal medicine (Hyeolbuchukeo-tang or Xuefu Zhuyu decoction) for treating primary dysmenorrhoea: protocol for a systematic review of randomised controlled trials

Journal:	*BMJ Open*
Manuscript ID	bmjopen-2016-015056.R1
Article Type:	Protocol
Date Submitted by the Author:	22-Mar-2017
Complete List of Authors:	Jo, Junyoung; Conmaul Hospital of Korean Medicine, Korean Obstetrics and Gynecology<br>Leem, Jungtae; Kyung Hee University, Department of Clinical Korean Medicine<br>Lee, Jin Moo; Kyung Hee University Korean Medicine Hospital at Gangdong , Department of Korean Obstetrics & Gynecology<br>PARK, KYOUNG SUN; Kyung Hee University, Korean Medicine Obstetrics & Gynecology
**Primary Subject Heading**:	Obstetrics and gynaecology
Secondary Subject Heading:	Complementary medicine, Evidence based practice
Keywords:	dysmenorrhoea, systematic review, Herbal medicine < THERAPEUTICS, protocol

SCHOLARONE™
Manuscripts

For peer review only

Herbal medicine (Hyeolbuchukeo-tang or Xuefu Zhuyu decoction) for treating primary dysmenorrhoea: protocol for a systematic review of randomised controlled trials

Junyoung Jo[1 †], Jungtae Leem[2,3 †], Jin Moo Lee[4], Kyoung Sun Park[4*]

[1]Department of Korean Medicine Obstetrics & Gynecology, Conmaul Hospital of Korean Medicine, Seoul 06634, Republic of Korea

[2]Korean Medicine Clinical Trial Center, Kyung Hee University Korean Medicine Hospital, Seoul 02447, South Korea

[3]Department of Clinical Korean Medicine, Graduate School, Kyung Hee University, 26 Kyungheedae-ro, Dongdaemun-gu, Seoul, 02447, South Korea

[4]Department of Korean Medicine Obstetrics & Gynecology, Kyung Hee University, 26 Kyungheedae-ro, Dongdaemun-gu, Seoul, 02447, South Korea

† equal contributors

* Correspondence: Kyoung Sun Park, M.D. (DKM), PhD

Department of Korean Medicine Obstetrics & Gynecology

Kyung Hee University

Seoul, Republic of Korea

Tel: 82-2-440-6146

Fax: 82-2- 440-7143

E-mail: lovepks0116@gmail.com

For peer review only

For peer review only - http://bmjopen.bmj.com/site/about/guidelines.xhtml

ABSTRACT

Introduction:

Primary dysmenorrhoea is menstrual pain without pelvic pathology and is the most common gynaecologic condition in women. Xuefu Zhuyu decoction (XZD) or Hyeolbuchukeo-tang, a traditional herbal formula, has been used as a treatment for primary dysmenorrhoea. The purpose of this study is to assess the current published evidence regarding XZD as treatment for primary dysmenorrhoea.

Methods and analysis:

The following databases will be searched from their inception until April, 2017: MEDLINE (via PubMed), Allied and Complementary Medicine Database (AMED), EMBASE, The Cochrane Library, six Korean Medical Databases (Korean Studies Information Service System, DBPIA, Oriental Medicine Advanced Searching Integrated System, Research Information Service System, Korea Med, and the Korean Traditional Knowledge Portal), three Chinese Medical Databases [China National Knowledge Infrastructure (CNKI), Wan Fang Database, and Chinese Scientific Journals Database (VIP)], and one Japanese medical database (CiNii). Randomised clinical trials (RCTs) that will be included in this systematic review comprise those that used XZD or modified XZD. The control groups in the RCTs include no treatment, placebo, conventional medication, or other treatments. Trials testing XZD as an adjunct to other treatments, as well as studies where the control group received the same treatment as the intervention group will be also included. Data extraction and risk of bias assessments will be performed by two independent reviewers. The risk of bias will be assessed with the Cochrane risk of bias tool. All statistical analyses will be conducted using Review Manager software (RevMan V.5.3.0).

Ethics and dissemination:

This systematic review will be published in a peer-reviewed journal. The review will also be disseminated electronically and in print. The review will benefit patients and practitioners in the fields of traditional and conventional medicine.

Trial registration number: CRD42016050447 in PROSPERO 2016

For peer review only

For peer review only - http://bmjopen.bmj.com/site/about/guidelines.xhtml

Strength and limitations of the present study protocol

- Our review will provide useful and novel information for patients, policymakers, and practitioners.
- To avoid language bias, the Chinese, Korean, and Japanese databases will be searched.
- Our systematic review will describe a comprehensive and objective assessment of the safety and effectiveness of Hyeolbuchukeo-tang/Xuefu Zhuyu decoction as treatment for primary dysmenorrhoea patients.
- We will assess the methodological and reporting quality of included studies with CONSORT extension for herbal medicine.
- One major limitation of our study protocol is that many of the included trials may have poor methodological quality or include insufficient explanation. This limitation compromises the accurate assessment of the quality of these clinical trials and their effect size. In addition, it means that insufficient information is available for future clinical trial protocol development.

1. Introduction

Primary dysmenorrhoea is a common complaint that refers to painful menstrual cramps in the lower abdominal region during menstruation in the absence of an identifiable pathological condition among menstruating women.[1] Due to the different definitions of the condition, and the lack of standard methods for assessing the severity of dysmenorrhoea, prevalence estimates vary between 45 and 95% of menstruating women.[2] Dysmenorrheic pain has been reported to be the primary cause of recurrent short-term school or work absenteeism among young women of childbearing age.[3] Further, dysmenorrheic pain has an immediate negative impact on quality of life, for up to a few days every month. Women with primary dysmenorrhoea have a significantly reduced quality of life, poorer mood, and poorer sleep quality during menstruation compared with women who do not report dysmenorrhoea.[3]

Non-steroidal anti-inflammatory drugs (NSAIDs) are considered the primary treatment for primary dysmenorrhoea but the quality of the evidence is low mainly due to poor reporting of study methods. In addition, NSAIDs commonly cause adverse effects, including indigestion, headaches, and drowsiness.[4] Therefore, many women also seek alternative therapies to manage their menstrual discomfort including heating pads for cramps,

For peer review only - http://bmjopen.bmj.com/site/about/guidelines.xhtml

For peer review only

transcutaneous electric nerve stimulation, Chinese herbal medicine (CHM), and acupuncture.[3 5-7] A Cochrane review suggested that CHM was promising for managing primary dysmenorrhoea, although the quality of the included studies was poor.[5] However, the review included all types of CHM and is outdated, requiring another study that focuses on a specific type of CHM.

In traditional Chinese Medicine or Korean Medicine, the main factor causing menstrual abdominal pain is blood stagnation.[8] Xuefu Zhuyu decoction (XZD) or Hyeolbuchukeo-tang was the most frequent formula used in the blood stasis researches in Korea.[9] Several systematic reviews regarding other CHM such as Danggui Shaoyao San[10], Shaofu Zhuyu decoction[11] or Gyejibongneyonghwan[12] have already been published or planned. However, no systematic review regarding XZD in primary dysmenorrhoea has been planned or published yet. Therefore, in this review, we will investigate current evidence related to the effectiveness of XZD or Hyeolbuchukeo-tang, a traditional herbal formula, as a treatment for primary dysmenorrhoea.

2. Materials and Methods

Study registration

The protocol for this systematic review has been registered on PROSPERO 2016 under the number CRD42016050447.

Data sources

The following databases will be searched from inception to April, 2017: Medline (via PubMed), EMBASE (via OVID), the Cochrane Central Register of Controlled Trials (CENTRAL), Allied and Complementary Medicine Database (AMED) and Cumulative Index to Nursing and Allied Health Literature (CINAHL). We will also search six Korean medical databases [Oriental Medicine Advanced Searching Integrated System (OASIS), Korean Traditional Knowledge Portal (KTKP), Korean Studies Information Service System (KISS), Research Information Service System (RISS), KoreaMed, and DBpia], three Chinese databases [China National Knowledge Infrastructure Database (CNKI), Wanfang, and Chinese Scientific Journals Database (VIP)], and one Japanese medical database (CiNii). We will also search conference proceedings of relevant journals and conduct hand searching. Clinical trial registries will also be searched. The search term will be composed of the disease term part (e.g., dysmenorrhoea, menstrual pain, painful menstruation, period pain, painful period, cramps, menstrual disorder, pelvic pain) and the intervention term part (e.g., Xuefu Zhuyu

For peer review only - http://bmjopen.bmj.com/site/about/guidelines.xhtml

granule/decoction/formula/tang/capsule/pill/tablet).

The search strategies that will be applied to the Medline database and CNKI are presented in online supplementary 1. Similar search strategies will be applied to the other databases. Study selection will be documented and summarized in a PRISMA-compliant flow chart (http://www.prisma-statement.org) (figure 1).[13]

Types of study

All prospective randomised controlled trials (RCTs) will be included. However, some Chinese articles do not describe the randomization method in detail but use only the word randomization (随机). We will include such articles but we will also assess the risk of bias as high if detailed randomization processes are not described. Some articles used inappropriate randomization processes, such as the tossing of a coin; we will exclude such articles. A crossover design clinical trial will be also included, but only the first phase data will be presented in the effect size tables and used in the meta-analysis. A pragmatic clinical trial will also be included, based on the agreement of two reviewers (JL, JJ).

Type of participants

Patients with primary dysmenorrhoea will be considered in the systematic review. Dysmenorrhoea secondary to other pathologies such as uterine myoma, endometriosis, or infection will not be included in this review.

Type of interventions

Randomized studies of the XZD formula, either as the sole treatment or as an adjunct to other treatments which were applied in both groups (intervention and control groups) in the same manner, will be included. Trials comparing XZD formula with any type of control intervention will also be included. Control group intervention could be placebo XZD, no treatment, conventional medication, or other treatments. XZD is composed of 11 herbs. We will also include modified XZD, which contains less than 50% of modified herbs; if the proportion of modified herbs is more than 50%, inclusion of such compounds will be determined based on the agreement of two researchers. No language restrictions will be imposed. Hard copies of all articles will be obtained and read in full text.

For peer review only - http://bmjopen.bmj.com/site/about/guidelines.xhtml

Data extraction

Two authors (JJ and JL) will perform the data extraction and quality assessment using a predefined data extraction form. The form includes information pertaining to first author, study design, language of publication, country where the trial was conducted, clinical setting, diagnostic criteria, disease duration, number of participants allocated to each group, drop out number, treatment duration, dosage of XZD, pattern identification of the participants' comparison groups, outcome, outcome results, follow-up periods, adverse events associated with XZD, and composition of XZD. When studies report outcomes at more than one time point, a similar measurement point in other studies will be obtained for analysis. Any disagreement among the authors will be resolved by discussion among all of the authors. When the data are insufficient or ambiguous, JL will contact the corresponding authors by e-mail or telephone to request additional information or clarification.

Assessment of risk of bias in included studies

We will assess risk of bias in included studies according to risk of bias assessment tool in Cochrane Handbook.[14]

Risk of bias in included studies will be classified into three categories (low, unclear, and high) by two independent reviewers. We will assess selective reporting, incomplete outcome data, blinding of the participants and personnel, blinding of the outcome assessments, allocation concealment, random sequence generation, and other sources of bias.[14] Disagreements between the two reviewers will be resolved by final decision of the arbiter (KP).

Outcome measures

Primary outcomes

- Change in symptoms as indicated on a 100 mm visual analogue scale (VAS)
- Response rate: an overall reduction in symptoms (menstruation-related symptoms including dysmenorrhoea)

As most Chinese trials report outcomes based on a categorical assessment (e.g. 'markedly improved', 'improved', 'slightly better', or 'no effect'), we will evaluate the response rates by three different methods because variation in effectiveness evaluation creates variation in results: 1) We will classify the "no effect" category as non-responder and other categories as responder. For example, if a treatment group of 100 women are measured for intensity of pain using markedly improved (n = 30), moderately improved (n = 40), slightly

For peer review only - http://bmjopen.bmj.com/site/about/guidelines.xhtml

better (n = 20), or no reduction (n=10), then the number of women who report any reduction (n = 90) will be considered as the responder group and included in the meta-analysis as having experienced a reduction in pain (n = 90/100). 2) Improvement in symptoms by >50% will be classified as responder; an improvement of <50% will be classified as non-responder. If the criteria for categorical assessment are not described or are unmatched, that article will not be included in the analysis. 3) We will classify the categories "no change" and "worsening of symptoms" as non-responder; we will classify the category "shows improvement" as responder.

Secondary outcomes

- Quality of life as measured using validated questionnaires
- Adverse events

Data synthesis and analysis

In order to help researchers, the effect size of every outcome in each clinical trial will be presented for future clinical trial protocol development. Statistical analyses will be performed with the Review Manager program (Version 5.3 Copenhagen: The Nordic Cochrane Centre, The Cochrane Collaboration, 2014). Trials will be combined according to the type of intervention and type of outcome measure and/or control. Data will be pooled and expressed as mean differences (MD) or standardized mean difference (SMD) for continuous outcomes and risk ratio (RR) for dichotomous outcomes with 95% confidence intervals (CI) using fixed or random-effects models.

Dealing with missing data

As much as possible, we will analyze the data using an intention-to-treat (ITT) basis, and we will attempt to obtain missing data from the original investigators. If these attempts are not successful, we will not impute data for missing data; we will analyze only the available data.

Assessment and investigation of heterogeneity

Heterogeneity among studies will be assessed using χ^2 (chi-squared) test with a significance level of $P < 0.1$ and I^2 statistic.[15] The I^2 statistic indicates the proportion of variability among trials that is not explained by chance alone and we consider an I^2 value > 50% to indicate a substantial heterogeneity.[15,16] If substantial

For peer review only - http://bmjopen.bmj.com/site/about/guidelines.xhtml

For peer review only

heterogeneity is detected, we will explore sources of heterogeneity by performing subgroup analysis. If some factors (e.g., lack of included trials, large methodological and/or clinical difference among trials) are found, we will not conduct subgroup analysis or data synthesis, but report a narrative description of the included studies. Subgroup analyses will be attempted according to type of control (e.g., kind of medicine), taking into consideration the characteristics of the included studies.

Subgroup analysis

If a sufficient number of subgroup studies exist, subgroup analysis will be conducted to identify heterogeneity between subgroups. Subgroup analysis criteria are as follows: 1) duration or dosage level of herbal medicine treatment; 2) type of control intervention: placebo XZD, no treatment, or western medication; and 3) duration or severity of primary dysmenorrhea; 4) pattern identification according to TCM theory; 5) physical form of XZD, i.e., decoctions, granules, or pills.

Sensitivity analysis

Methodological and reporting quality of included studies will be assessed by the consolidated standards of reporting trials (CONSORT) extension for herbal interventions.[17] To identify the robustness of the meta-analysis result, sensitivity analysis will be conducted after excluding low quality trials. We will compare original and sensitivity meta-analysis results.

Assessment of reporting biases

When there are more than 10 trials in the analysis, reporting biases such as publication bias will be assessed by funnel plots. If asymmetry is suggested by a visual inspection, we will perform exploratory analyses using Egger's method.[15]

3. Discussion, Ethics and Dissemination

The purpose of our review is to assess the effectiveness and safety of XZD in women with primary dysmenorrhoea. Several systematic reviews of CHM have already been published.[5 10 11] Even though XZD is frequently used in primary dysmenorrhoea[18], no systematic reviews on the effects of XZD formula on primary dysmenorrhoea have been published. This systematic review will provide a summary of the current evidence

For peer review only - http://bmjopen.bmj.com/site/about/guidelines.xhtml

related to the effectiveness of XZD formula for the treatment of primary dysmenorrhoea. In particular, we will identify subtypes that are particularly useful for specific subgroups according to TCM theory or TCM pattern identification. We will also identify a range of dosages and modifications used to improve effectiveness in full review of this protocol. We know that most of the systematic reviews in the field of traditional medicine have drawn the conclusion that "there is some supporting evidence for the use of herbal medication but the methodological and reporting quality are both poor". We believe that the purpose of a systematic review is not simply the mathematical synthesis of existing clinical trial results, but also to offer detailed information relevant to clinical trial protocol development and clinical practice. Accordingly, we will show the effect size for all clinical trials to help researchers and physicians. Detailed information on the clinical trial regimens of XZD in primary dysmenorrhea will also provide an insight to researchers who are planning XZD clinical trials on this subject. We also anticipate finding predicting factors of treatment response by subgroup analysis. This evidence will also be useful to medical practitioners and patients in the field of women health care.

This systematic review does not need ethical approval because only published data will be included in our review. This systematic review will be published in a peer-reviewed journal. The review will also be disseminated electronically and in print. The results will be presented in international academic conference. The review will benefit patients and practitioners in the fields of traditional and conventional medicine.

Reference

1. Dawood MY. Primary dysmenorrhoea: advances in pathogenesis and management. *Obstetrics and gynecology* 2006;108(2):428-41. doi: 10.1097/01.AOG.0000230214.26638.0c [published Online First: 2006/08/02]
2. Proctor M, Farquhar C. Diagnosis and management of dysmenorrhoea. *BMJ (Clinical research ed)* 2006;332(7550):1134-8. doi: 10.1136/bmj.332.7550.1134 [published Online First: 2006/05/13]
3. Iacovides S, Avidon I, Baker FC. What we know about primary dysmenorrhoea today: a critical review. *Human reproduction update* 2015;21(6):762-78. doi: 10.1093/humupd/dmv039 [published Online First: 2015/09/09]
4. Marjoribanks J, Ayeleke RO, Farquhar C, et al. Nonsteroidal anti-inflammatory drugs for dysmenorrhoea. *The Cochrane database of systematic reviews* 2015(7):Cd001751. doi: 10.1002/14651858.CD001751.pub3 [published Online First: 2015/08/01]

For peer review only - http://bmjopen.bmj.com/site/about/guidelines.xhtml

5. Zhu X, Proctor M, Bensoussan A, et al. Chinese herbal medicine for primary dysmenorrhoea. *The Cochrane database of systematic reviews* 2008(2):Cd005288. doi: 10.1002/14651858.CD005288.pub3 [published Online First: 2008/04/22]

6. Smith CA, Armour M, Zhu X, et al. Acupuncture for dysmenorrhoea. *The Cochrane database of systematic reviews* 2016;4:Cd007854. doi: 10.1002/14651858.CD007854.pub3 [published Online First: 2016/04/19]

7. Igwea SE, Tabansi-Ochuogu CS, Abaraogu UO. TENS and heat therapy for pain relief and quality of life improvement in individuals with primary dysmenorrhoea: A systematic review. *Complementary therapies in clinical practice* 2016;24:86-91. doi: 10.1016/j.ctcp.2016.05.001 [published Online First: 2016/08/10]

8. Park JS, Park S, Cheon CH, et al. Effects and safety of gyejibongnyeong-hwan on dysmenorrhoea caused by blood stagnation: a randomised controlled trial. *Evidence-based complementary and alternative medicine : eCAM* 2013;2013:424730. doi: 10.1155/2013/424730 [published Online First: 2013/11/06]

9. Park B, You S, Jung J, et al. Korean studies on blood stasis: an overview. *Evidence-based complementary and alternative medicine : eCAM* 2015;2015:316872. doi: 10.1155/2015/316872 [published Online First: 2015/03/31]

10. Lee HW, Jun JH, Kil KJ, et al. Herbal medicine (Danggui Shaoyao San) for treating primary dysmenorrhoea: A systematic review and meta-analysis of randomised controlled trials. *Maturitas* 2016;85:19-26. doi: 10.1016/j.maturitas.2015.11.013 [published Online First: 2016/02/10]

11. Lee H, Choi TY, Myung CS, et al. Herbal medicine (Shaofu Zhuyu decoction) for treating primary dysmenorrhoea: A systematic review of randomised clinical trials. *Maturitas* 2016;86:64-73. doi: 10.1016/j.maturitas.2016.01.012 [published Online First: 2016/02/29]

12. Lee JA, Park S, Jung J, et al. Herbal medicine (Gyejibongneyong-hwan) for treating primary dysmenorrhoea: a protocol for a systematic review of randomised controlled trials. *BMJ open* 2016;6(9):e011071. doi: 10.1136/bmjopen-2016-011071 [published Online First: 2016/09/30]

13. Liberati A, Altman DG, Tetzlaff J, et al. The PRISMA statement for reporting systematic reviews and meta-analyses of studies that evaluate health care interventions: explanation and elaboration. *Annals of internal medicine* 2009;151(4):W65-94. [published Online First: 2009/07/23]

14. Higgins JPT, Altman DG. "Chapter 8: assessing risk of bias in included studies," in Cochrane Handbook for Systematic Reviews of Interventions Version 5.1.0, J. Higgins and S. Green, Eds., The Cochrane Collaboration, 2011, http://www.cochrane-handbook.org. 2011 [

15. Deeks JJ, Higgins JPT, Altman DG. "Chapter 9: analyzing data and undertaking meta-analyses," in Cochrane Handbook for Systematic Reviews of Interventions Version 5.1.0, J. Higgins and S. Green, Eds., The Cochrane Collaboration, 2011, http://www.cochrane-handbook.org.

For peer review only - http://bmjopen.bmj.com/site/about/guidelines.xhtml

2011 [
16. Higgins JP, Thompson SG. Quantifying heterogeneity in a meta-analysis. *Stat Med* 2002;21(11):1539-5158.
17. Gagnier JJ, Boon H, Rochon P, et al. Recommendations for reporting randomised controlled trials of herbal interventions: Explanation and elaboration. *Journal of clinical epidemiology* 2006;59(11):1134-49. doi: 10.1016/j.jclinepi.2005.12.020 [published Online First: 2006/10/10]
18. Jia W, Wang X, Xu D, et al. Common traditional Chinese medicinal herbs for dysmenorrhoea. *Phytotherapy research : PTR* 2006;20(10):819-24. doi: 10.1002/ptr.1905 [published Online First: 2006/07/13]

AUTHORS' CONTRIBUTIONS

The study was conceptualized by JJ. The protocol was drafted by JJ and J Leem. The search strategy was developed by J Leem and JJ. J Lee and KP revised the manuscript. All authors have read and approved the final manuscript.

FUNDING AND ACKNOWLEDGEMENTS

This study is supported by the Traditional Korean Medicine R&D program that is funded by the Ministry of Health & Welfare through the Korea Health Industry Development Institute (KHIDI, grant HB16C0018).

COMPETING INTERESTS

The authors declare no competing interests.

PROVENANCE AND PEER REVIEW

Not commissioned; externally peer reviewed.

For peer review only

For peer review only - http://bmjopen.bmj.com/site/about/guidelines.xhtml

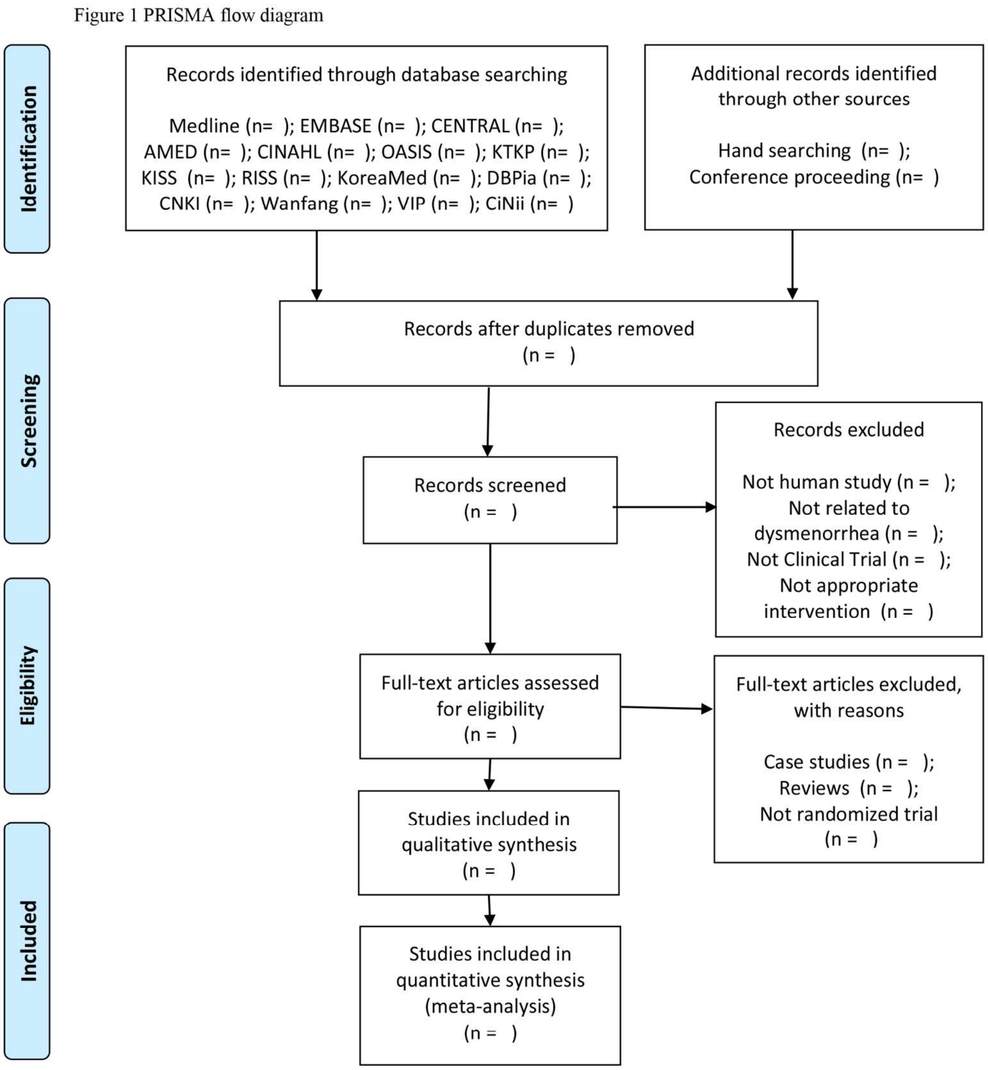

Figure 1 PRISMA Flow Diagram

197x223mm (300 x 300 DPI)

Supplementary 1 Search strategies

[MEDLINE(Pubmed) Search Strategy]

#1 "Menstruation Disturbances"[Mesh]

#2 Dysmenorrhea [TIAB]

#3 Pain, Menstrual [TIAB]

#4 Menstrual Pain [TIAB]

#5 Menstrual Pains [TIAB]

#6 Pains, Menstrual [TIAB]

#7 Menstruation, Painful [TIAB]

#8 Menstruations, Painful [TIAB]

#9 Painful Menstruation [TIAB]

#10 Painful Menstruations [TIAB]

#11 Period pain [TIAB]

#12 Painful period [TIAB]

#13 Cramps [TIAB]

#14 Menstrual Disorder [TIAB]

#15 Pelvic pain [TIAB]

#16 1-15/or

#17 Xuefu Zhuyu [TIAB]

#18 “Xuefu Zhuyu Granule” [TIAB]

#19 “Xuefu Zhuyu Decoction” [TIAB]

#20 “Xuefu Zhuyu Formula” [TIAB]

#21 “Xuefu Zhuyu Tang” [TIAB]

For peer review only

#22 Xuefu Zhuyu Capsule [TIAB]

#23 “Xuefu Zhuyu Pill” [TIAB]

#24 “Xuefu Zhuyu Tablet” [TIAB]

#25 “Xuefu Zhuyu Oral Liquid” [TIAB]

#26 Xue fu Zhu yu [TIAB]

#27 “Xue fu Zhu yu Granule” [TIAB]

#28 “Xue fu Zhu yu Decoction” [TIAB]

#29 “Xue fu Zhu yu Formula” [TIAB]

#30 “Xue fu Zhu yu Tang” [TIAB]

#31 Xue fu Zhu yu Capsule [TIAB]

#32 “Xue fu Zhu yu Pill” [TIAB]

#33 “Xue fu Zhu yu Tablet” [TIAB]

#34 “Xue fu Zhu yu Oral Liquid” [TIAB]

#35 “Hyeolbuchukeo-tang” [TIAB]

#36 “Hyulbuchuko-tang” [TIAB]

#37 “Hyulboochucke-tang” [TIAB]

#38 “Hyulbuchookau-tang” [TIAB]

#39 17-38/or

#40 #16 and #39

[CNKI Search Strategy]

#1 痛经 OR 原发性痛经 OR 月经失调 OR 月经困难 OR 月经紊乱 OR 经行腹痛 OR 经痛 OR 月经痛 OR 痛性痉挛 OR 骨盆痛

For peer review only

Search in result

#2 血府逐瘀 OR 血府逐瘀汤 OR 血府逐瘀颗粒 OR血府逐瘀方 OR 血府逐瘀汤 OR 血府逐瘀胶囊 OR 血府逐瘀丸 OR 血府逐瘀片

For peer review only

PRISMA-P 2015 Checklist

This checklist has been adapted for use with protocol submissions to *Systematic Reviews* from Table 3 in Moher D et al: Preferred reporting items for systematic review and meta-analysis protocols (PRISMA-P) 2015 statement. *Systematic Reviews* 2015 **4**:1

Section/topic	#	Checklist item	Information reported		Line number(s)
			Yes	No	
**ADMINISTRATIVE INFORMATION**					
**Title**					
Identification	1a	Identify the report as a protocol of a systematic review	☐		(p.1) 1-2
Update	1b	If the protocol is for an update of a previous systematic review, identify as such			Not update for previous review
**Registration**	2	If registered, provide the name of the registry (e.g., PROSPERO) and registration number in the Abstract	☐		(p.2) 53
**Authors**					
Contact	3a	Provide name, institutional affiliation, and e-mail address of all protocol authors; provide physical mailing address of corresponding author	☐		(p.1)4-23
Contributions	3b	Describe contributions of protocol authors and identify the guarantor of the review	☐		(p.11)304-307
**Amendments**	4	If the protocol represents an amendment of a previously completed or published protocol, identify as such and list changes; otherwise, state plan for documenting important protocol amendments			Not amendment for previous review
**Support**					
Sources	5a	Indicate sources of financial or other support for the review	☐		(p.11)309-311
Sponsor	5b	Provide name for the review funder and/or sponsor	☐		(p.11)309-311
Role of sponsor/funder	5c	Describe roles of funder(s), sponsor(s), and/or institution(s), if any, in developing the protocol			No roles exist
**INTRODUCTION**					

BioMed Central The Open Access Publisher

For peer review only - http://bmjopen.bmj.com/site/about/guidelines.xhtml

Section/topic	#	Checklist item	Information reported Yes	Information reported No	Line number(s)
**Rationale**	6	Describe the rationale for the review in the context of what is already known	☐		(p.3-4)70-93
**Objectives**	7	Provide an explicit statement of the question(s) the review will address with reference to participants, interventions, comparators, and outcomes (PICO)	☐		(p.5-7)127-178
**METHODS**					
**Eligibility criteria**	8	Specify the study characteristics (e.g., PICO, study design, setting, time frame) and report characteristics (e.g., years considered, language, publication status) to be used as criteria for eligibility for the review	☐		(p.5)117-124
**Information sources**	9	Describe all intended information sources (e.g., electronic databases, contact with study authors, trial registers, or other grey literature sources) with planned dates of coverage	☐		(p.4-5)100-115
**Search strategy**	10	Present draft of search strategy to be used for at least one electronic database, including planned limits, such that it could be repeated	☐		(p.4-5)100-115
STUDY RECORDS					
Data management	11a	Describe the mechanism(s) that will be used to manage records and data throughout the review	☐		(p.6)141-150
Selection process	11b	State the process that will be used for selecting studies (e.g., two independent reviewers) through each phase of the review (i.e., screening, eligibility, and inclusion in meta-analysis)	☐		(p.6)141-150
Data collection process	11c	Describe planned method of extracting data from reports (e.g., piloting forms, done independently, in duplicate), any processes for obtaining and confirming data from investigators	☐		(p.6)141-150
**Data items**	12	List and define all variables for which data will be sought (e.g., PICO items, funding sources), any pre-planned data assumptions and simplifications	☐		(p.6-7)160-178
**Outcomes and prioritization**	13	List and define all outcomes for which data will be sought, including prioritization of main and additional outcomes, with rationale	☐		(p.6-7)160-178
**Risk of bias in individual studies**	14	Describe anticipated methods for assessing risk of bias of individual studies, including whether this will be done at the outcome or study level, or both; state how this information will be used in data synthesis	☐		(p.6)151-158
DATA					
**Synthesis**	15a	Describe criteria under which study data will be quantitatively synthesized	☐		(p.7)180-187
	15b	If data are appropriate for quantitative synthesis, describe planned summary measures, methods of handling data, and methods of combining data from studies, including any planned exploration	☐		(p.7-8)180-202

BioMed Central
The Open Access Publisher

For peer review only

For peer review only - http://bmjopen.bmj.com/site/about/guidelines.xhtml

3

Section/topic	#	Checklist item	Information reported		Line number(s)
			Yes	No	
		of consistency (e.g., I^2, Kendall's tau)			
	15c	Describe any proposed additional analyses (e.g., sensitivity or subgroup analyses, meta-regression)	☐		(p.8)204-215
	15d	If quantitative synthesis is not appropriate, describe the type of summary planned	☐		(p.7-8)194-202
**Meta-bias(es)**	16	Specify any planned assessment of meta-bias(es) (e.g., publication bias across studies, selective reporting within studies)	☐		(p.8)204-220
**Confidence in cumulative evidence**	17	Describe how the strength of the body of evidence will be assessed (e.g., GRADE)		☐	Not Applicable

For peer review only

BioMed Central
The Open Access Publisher
